# Retinoic Acid and Calcitriol Protect Mouse Primordial Follicles from Cyclophosphamide Treatment-Induced Apoptosis

**DOI:** 10.3390/antiox15010068

**Published:** 2026-01-04

**Authors:** Sihui He, Xiaodan Zhang, Wenjun Zhou, Ye Chen, Fengxin Liu, Weiyong Wang, Hongwei Wei, Yan Du, Meijia Zhang

**Affiliations:** The Innovation Centre of Ministry of Education for Development and Diseases, The Second Affiliated Hospital, School of Medicine, South China University of Technology, Guangzhou 510006, China; 15626459393@163.com (S.H.); zxdscut@scut.edu.cn (X.Z.); lovelychou@126.com (W.Z.); chenye45@foxmail.com (Y.C.); 20202521056@m.scnu.edu.cn (F.L.); wangwy@kust.edu.cn (W.W.); 18390237338@163.com (H.W.); 18763811882@163.com (Y.D.)

**Keywords:** cyclophosphamide, retinoic acid, calcitriol, primordial follicle, apoptosis

## Abstract

Chemotherapy causes primordial follicle apoptosis, resulting in premature ovarian insufficiency (POI) and infertility. In this study, we found that intraperitoneal injection of retinoic acid (RA) and calcitriol partially reversed the cyclophosphamide and doxorubicin treatment-induced decrease in primordial follicles in neonatal mouse ovaries. Furthermore, RA and calcitriol co-treatment reversed cyclophosphamide treatment-induced PI3K/Akt activity and FOXO3a nuclear export in the oocytes within primordial follicles, suggesting that the oocyte transcriptional activity was decreased, which in turn reduced the binding of chemotherapeutic drugs to DNA. Consistent with these findings, RA and calcitriol co-treatment reversed cyclophosphamide treatment-induced changes in reactive oxygen species (ROS), DNA damage response proteins (γH2AX, p-CHK2, p-p53, PUMA, BAX, Cleaved Caspase-3, and cPARP), and antioxidant proteins (NRF2, HO-1, and GPX4). Moreover, RA and calcitriol co-treatment preserved fertility in cyclophosphamide-treated mice without impairing cyclophosphamide’s antitumor efficacy in MCF-7 tumor-bearing mice. Thus, RA and calcitriol protect mouse primordial follicles from cyclophosphamide treatment-induced apoptosis by inhibiting cyclophosphamide treatment-induced oocyte transcriptional activity and enhancing antioxidant capacity. Our results suggest a potential strategy for preserving ovarian reserve during chemotherapy in female cancer patients.

## 1. Introduction

Cancer incidence is rising annually, with an observable trend toward younger ages [1]. This shift is driven by various factors [2], such as environmental pollution [3], drug residues [4], sleep deprivation [5], smoking prevalence [6], and unhealthy diets [7]. Chemotherapeutic drugs are widely used to improve the survival rate of patients with various cancers [8]. Both cyclophosphamide and doxorubicin are widely used to treat many malignant tumors such as lymphoma, leukemia, and breast cancer [9,10,11]. Cyclophosphamide is metabolized by hepatic microsomal cytochrome P450 to its active metabolites, phosphoramide mustard and acrolein [12]. Phosphoramide mustard alkylates DNA at the N-7 position of guanine, resulting in intra- and inter-strand DNA cross-links that prevent DNA replication and tumor cell division [13,14]. Acrolein causes a rapid reduction in intracellular glutathione (GSH) and overall antioxidant capacity, leading to excessive production of reactive oxygen species (ROS) and oxidative stress [9]. Acrolein also disrupts mitochondrial integrity and function, resulting in pro-apoptotic protein release and ATP depletion [15]. Doxorubicin (DOX) damages cancer cells by binding to topoisomerase II (TOP2) to form a covalent complex with DNA (DOX–TOP2–DNA) [16]. This complex inhibits DNA replication and transcription, resulting in DNA double-strand breaks (DSBs) and tumor cell apoptosis [17].

In the past few decades, the overall 5-year survival rates of all cancers combined have increased substantially, exceeding 90% for childhood acute lymphocytic leukemia and early-stage breast cancer [18]. However, most chemotherapeutic drugs, including cyclophosphamide and doxorubicin, cause massive apoptosis of primordial follicles, leading to premature ovarian insufficiency (POI) and female infertility [19,20,21]. Cancer survivors strongly seek to restore their ovarian function to maintain endocrine homeostasis and fertility [22]. Current fertility preservation strategies include oocyte and embryo cryopreservation for adult cancer patients [23] and ovarian tissue cryopreservation for prepubescent cancer patients before chemotherapy [24]. Subsequent assisted reproductive technologies are still needed to achieve fertility, including in vitro activation (IVA), in vitro fertilization (IVF), and/or embryo transfer [19,25]. All of these processes limit the final success rate. Moreover, the surgical removal of normal ovarian tissues from cancer patients results in a significant psychological burden, which will induce immunosenescence that decreases antitumor immunity [26,27]. Therefore, it is necessary to develop non-invasive strategies to protect ovarian function in situ during chemotherapy.

The follicles in the mammalian ovary are divided into two types: primordial and growing follicles. The primordial follicle pool, about 1–2 million oocytes in size, is established around the time of birth and is non-renewable [28]. In each wave, only a few primordial follicles develop into growing follicles, and they are ultimately depleted through ovulation or atresia [29]. The remaining primordial follicles are in a dormant state to maintain female reproductive lifespan [30]. Thus, the essence of protecting ovarian function is to protect the primordial follicles.

Retinoic acid (RA), also known as all-trans retinoic acid (ATRA), is the main active form of vitamin A [31]. RA can promote oocyte maturation [32,33], fertilization [34], and early embryonic development [35] in various mammalian species. RA works through the heterodimeric complexes of retinoic acid receptors (RARs) and retinoid X receptors (RXRs) [36]. RARs, including RARα, RARβ, and RARγ, are expressed in both human and mouse ovaries [37,38,39]. RXR can also form heterodimeric complexes with the vitamin D receptor (VDR) [40]. VDR is activated by calcitriol (1,25-dihydroxy vitamin D3), the active form of vitamin D [41]. Calcitriol/VDR is also crucial for follicular development [42]. Both RA and calcitriol have antioxidant, anti-inflammatory, and antitumor properties [42,43,44]. Therefore, we hypothesize that RA and calcitriol can maintain the quiescence of primordial follicle oocytes to reduce cyclophosphamide treatment-induced primordial follicle apoptosis, thereby preserving the primordial follicle reserve.

In the present study, RA and calcitriol co-treatment partially reversed cyclophosphamide treatment-induced primordial follicle apoptosis and fertility decrease in mice by inhibiting FOXO3a nuclear export and transcriptional activity in primordial follicle oocytes. Furthermore, RA and calcitriol preserved ovarian reserve without impairing cyclophosphamide’s antitumor efficacy in MCF-7 tumor-bearing mice. Therefore, our results suggest a promising non-invasive strategy for preserving ovarian reserve during chemotherapy in female cancer patients.

## 2. Methods

### 2.1. Animals and Chemicals

3- and 8-week-old ICR mice were purchased from the Guangdong Medical Laboratory Animal Center (Guangzhou, China), and 6–8-week-old female BALB/c-Nude mice were provided by GemPharmatech Co., Ltd. (Nanjing, China). All mice were housed in the animal facility of South China University of Technology under standardized conditions, with 20–24 °C, 50–70% relative humidity, and a 12-h/12 h light/dark cycle. The neonatal mice were obtained from the breeding of adult ICR mice, and the day of birth was designated as 0.5 days postpartum (dpp). Unless otherwise stated, all reagents were provided by Sigma-Aldrich (St. Louis, MO, USA).

### 2.2. Cell Culture

MCF-7 human breast cancer cells were purchased from Pricella Biotechnology Co., Ltd. (Wuhan, China), and cultured in RPMI-1640 medium (Gibco, Grand Island, NY, USA) containing 10% heat-inactivated fetal bovine serum (FBS, Thermo Fisher Scientific, Waltham, MA, USA) and 1% penicillin–streptomycin within an incubator at 37 °C with 5% CO_2_. Cells were passaged when they reached 90% confluence and were collected with 0.25% trypsin (Thermo Fisher Scientific) for experiments when they were in a logarithmic growth phase [45].

### 2.3. Animal Model and Treatments

Neonatal mice were intraperitoneally injected with cyclophosphamide (75 mg/kg), doxorubicin (10 mg/kg) or an equal volume of PBS on 5 dpp. At the same time, RA (0, 5, 15, 25 mg/kg), calcitriol (Cal; 0, 25, 50, 75 ng/kg), 25 mg/kg RA + 50 ng/kg calcitriol (Vit) or an equal volume of DMSO was intraperitoneally injected into the mice on 3, 5, and 7 dpp. Ovaries were collected from mice on 6 dpp for Western blotting and immunofluorescence staining, and were collected from mice on 8 dpp for follicle counting [46,47].

Adolescent mice (mice on 21 dpp as day 1) were intraperitoneally injected with cyclophosphamide (75 mg/kg) or an equal volume of PBS on day 5, and with Vit (25 mg/kg RA + 50 ng/kg calcitriol) or an equal volume of DMSO on days 1, 3, 5, 7, and 9. Six mice per group were sacrificed on days 8 and 26, and the ovaries were collected for follicle counting. The remaining females (*n* = 18/group) were mated with fertility-proven males from day 61 to day 256 [48,49].

The 6–8-week-old female BALB/c-Nude mice were subcutaneously injected with 1.4 × 10^7^ MCF-7 cells in 0.1 mL PBS into the left mammary fat pad. The drug treatment was started (designated as day 1) when the tumor volume of each mouse reached approximately 100 mm^3^. MCF-7 tumor-bearing mice were randomly divided into four groups: control, cyclophosphamide, cyclophosphamide + Vit, and Vit. MCF-7 tumor-bearing mice were intraperitoneally injected with cyclophosphamide (75 mg/kg) or an equal volume of PBS on day 3, 10, and 17, and with Vit (25 mg/kg RA + 50 ng/kg calcitriol) or an equal volume of DMSO on days 1, 3, 5, 8, 10, 12, 15, 17, and 19.

The tumor sizes were measured every 3 days, and the tumor volume was determined using the formula tumor volume (mm^3^) = LW^2^/2, where L represents the length, and W indicates the width. On day 21, BALB/c mice were euthanized by neck dislocation. Tumors and ovaries were immediately removed for tumor inhibition analysis and follicle counting, respectively [45]. The tumor growth inhibition rate was assessed according to the following formula: inhibition rate (%) = (1 − tumor volume of treatment group/tumor volume of control group) × 100%.

### 2.4. Histological Analysis and Follicle Counting

After paraformaldehyde fixing, graded ethanol dehydrating, and paraffin embedding, ovarian samples were sectioned serially at 5 µm as reported before [30], and then hematoxylin (Solarbio, Beijing, China) was used to stain these sections. To assess the total quantity of primordial follicles per ovary, primordial follicles were counted in every fifth section, with the total number calculated by multiplying the count by a correction factor of 5. Primary, secondary, and antral follicles (growing follicles) were counted in consecutive sections. Only non-overlapping follicles containing visible oocyte nuclei were counted to avoid double-counting. All sections were assessed by two independent individuals blinded to all groups.

### 2.5. Immunofluorescence

Immunofluorescence staining was performed following the protocol described in the previous study [29]. Briefly, after dewaxing and rehydrating, the ovarian sections underwent antigen retrieval with sodium citrate buffer, blockade with donkey serum, and then incubation with primary antibodies (Supplementary Table S1) at 4 °C overnight. The sections were then treated with secondary antibodies conjugated to Alexa Fluor 488 or 555 (Thermo Fisher Scientific) for 1 h at 37 °C. Finally, the sections were stained with 4′,6-diamidino-2-phenylindole (DAPI) for 5 min, and then treated with anti-fluorescence quenching mounting medium (Ruitaibio, Beijing, China). All sections were imaged using an LSM 800 confocal microscope (Carl Zeiss, Oberkochen, Germany) under the same imaging parameters. ZEN software (Carl Zeiss, Version 3.1) was used to analyze and quantify fluorescence intensity. The mean value of the five largest sections in each ovary was considered one independent replicate.

### 2.6. Isolation of Oocytes from Neonatal Mice

The collected ovaries were digested with 0.25% trypsin (Thermo Fisher Scientific) for 10 min at 37 °C and then were terminated via the addition of 10% FBS (Thermo Fisher Scientific) in pre-warmed M2 medium (Thermo Fisher Scientific). Oocytes were released from the follicles. Then, oocytes were collected from the cell suspension using mouth-operated glass pipettes under a stereomicroscope, washed thoroughly, and placed in pre-warmed M2 medium (Thermo Fisher Scientific) droplets for later use.

### 2.7. ROS Staining of Oocytes

The ROS levels in oocytes were detected following the ROS detection kit (Beyotime, Beijing, China) [50]. Briefly, oocytes were incubated in pre-warmed M2 medium (Thermo Fisher Scientific) with 10 μM 2′,7′-dichlorodihydrofluorescein diacetate (DCFH-DA, Beyotime) for 30 min at 37 °C in the dark, and then washed three times in pre-warmed M2 medium. Subsequently, the oocytes were transferred to cell culture dishes (NEST, Beijing, China) and imaged under the same parameters using an LSM 800 confocal microscope (Carl Zeiss).

### 2.8. TUNEL Staining

Follicle apoptosis in the ovaries was detected by the Click-iT Plus TUNEL Assay (Thermo Fisher Scientific) [50]. Briefly, dewaxed and rehydrated ovarian sections were permeabilized with proteinase K for 30 min at room temperature. After two washes with PBS, the sections were incubated with the TUNEL reaction mixture for 1 h at 37 °C in the dark. Subsequently, nuclei were stained with DAPI (Beyotime) for visualization. All sections were imaged with an LSM 800 confocal microscope (Carl Zeiss) under the same parameters.

### 2.9. Western Blotting

6–8 ovaries per replicate were collected for protein extraction as reported before [29]. Briefly, the ovarian samples were lysed on ice, and proteins were collected to detect the concentration. Equal quantities of protein (20 μg) were combined with SDS loading buffer (Cwbio, Beijing, China) and denatured by heating at 95 °C for 10 min and loaded into a 5% stacking gel and a 10% separating gel for electrophoresis. After that, the proteins were transferred to polyvinylidene difluoride (PVDF) membranes. The membranes were then blocked with 5% skim milk, followed by an overnight incubation with primary antibodies (Supplementary Table S1) at 4 °C and a 1 h incubation with anti-mouse or anti-rabbit IgG (1:5000, ZSGB-BIO, Beijing, China) at room temperature. Protein bands were detected using a chemiluminescent substrate (NCM Biotech, Suzhou, China) and captured with a chemiluminescence imaging system (Tanon, Shanghai, China). All protein band densities were measured using ImageJ software (version 1.4.3.67; NIH Image, Bethesda, MD, USA), using GAPDH as the internal control.

### 2.10. RNA-Sequencing

Neonatal mice were intraperitoneally injected with cyclophosphamide (75 mg/kg), doxorubicin (10 mg/kg), or an equal volume of PBS on 5 dpp. At the same time, RA (5, 15, 25 mg/kg), calcitriol (Cal; 25, 50, 75 ng/kg), 25 mg/kg RA + 50 ng/kg calcitriol (Vit), or an equal volume of DMSO was intraperitoneally injected into the mice on 3 and 5 dpp. Ovaries were collected from mice on 6 dpp, and preserved in sample buffer before being submitted for RNA extraction and subsequent RNA sequencing at Kidio Biotechnology Co., Ltd. (Guangzhou, China). The analysis was performed using Metascape (https://metascape.org) and Omicstudio (https://www.omicstudio.cn).

### 2.11. Statistical Analysis

All experiments were performed with at least three independent replicates. Data are displayed as the mean ± standard deviation (SD) and analyzed by one-way ANOVA with Tukey’s multiple-comparison test or a two-tailed unpaired Student’s *t*-test. Statistical analyses were performed using the GraphPad Prism software (version 8.0.1, La Jolla, CA, USA).

## 3. Results

### 3.1. RA and Calcitriol Reduce Cyclophosphamide and Doxorubicin Treatment-Induced Primordial Follicle Loss in Mice

Analysis of scRNA-seq data (GSE263836) revealed that RARs were predominantly expressed in mouse oocytes of primordial follicles (Figure S1). The injection of RA or calcitriol in neonatal mice decreased the number of growing follicles in the ovaries (Figure S2), and the most effective doses were 25 mg/kg and 50 ng/kg for RA and calcitriol, respectively (Figure S2). This is consistent with our recent studies that both RA and calcitriol inhibit mouse primordial follicle activation. Consistent with previous studies [47,48], the injection of a single dose of 75 mg/kg cyclophosphamide or 10 mg/kg doxorubicin to neonatal mice resulted in the loss of a large number of primordial follicles, along with an increase in the number of growing follicles in the cyclophosphamide treatment group (Figure S3).

**Figure 1:**
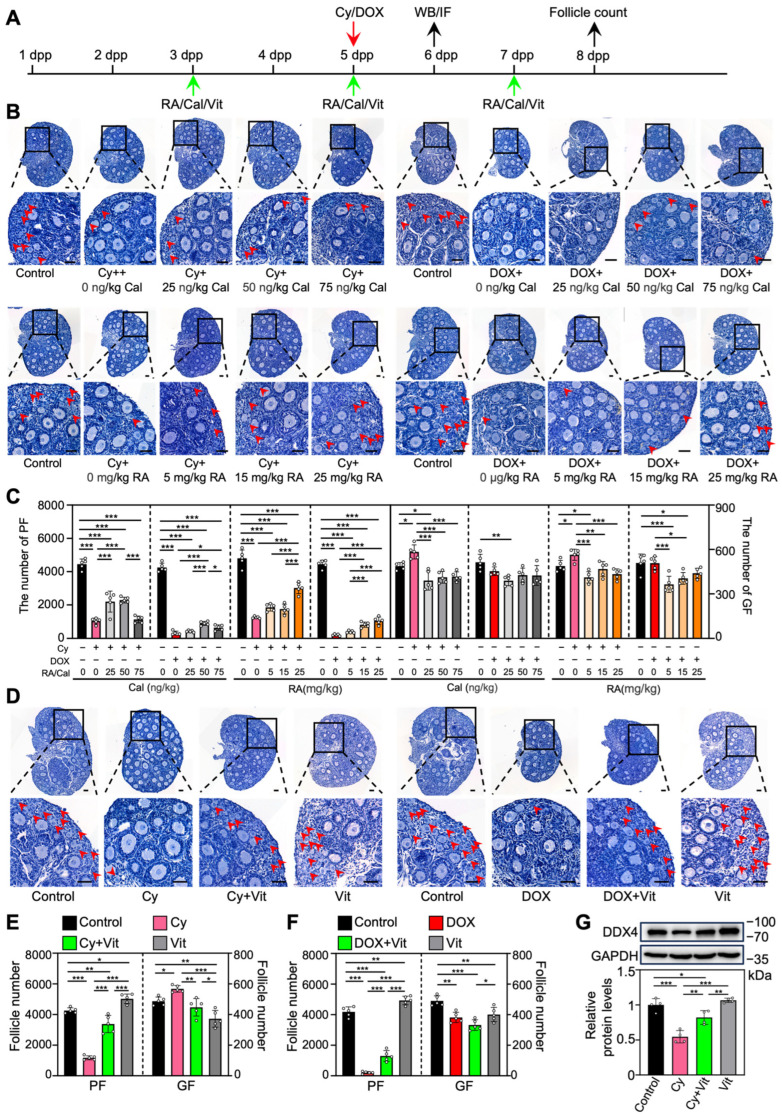
Effects of retinoic acid and calcitriol on cyclophosphamide or doxorubicin treatment-induced primordial follicle loss: (**A**) Experimental schema. Cyclophosphamide (Cy; 75 mg/kg) or doxorubicin (DOX; 10 mg/kg) was intraperitoneally injected into the mice on 5 dpp (red arrow). At the same time, RA (0, 5, 15, 25 mg/kg), calcitriol (Cal; 0, 25, 50, 75 ng/kg), or 25 mg/kg RA + 50 ng/kg calcitriol (Vit) was intraperitoneally injected into the mice on 3, 5, and 7 dpp (green arrows). Equal doses of PBS or DMSO were intraperitoneally injected into the mice as the corresponding controls. Ovaries were collected from mice on 6 dpp (black arrow) for Western blot analysis or immunofluorescence staining, and from mice on 8 dpp (black arrow) for follicle counting. (**B**–**F**) Ovarian morphological comparison (**B**,**D**) and the number of primordial and growing follicles (**C**,**E**,**F**) across various groups, *n* = 5, and each from 3 ovaries. The ovarian sections were hematoxylin-stained. Red arrowheads, primordial follicles. Scale bars, 50 µm. PF, primordial follicle; GF, growing follicle. (**G**) The comparison of DDX4 protein levels by Western blot across various groups, *n* = 4, and each from 6–8 ovaries. The representative images were displayed. Bars indicate the mean ± SD. Data were analyzed by one-way ANOVA followed by Tukey’s multiple-comparison test. * *p* < 0.05, ** *p* < 0.01, and *** *p* < 0.001.

To investigate the effect of RA and calcitriol on cyclophosphamide or doxorubicin treatment-induced primordial follicle loss, we intraperitoneally injected neonatal mice with cyclophosphamide or doxorubicin, and with RA and calcitriol before and after chemotherapy drug treatment (Figure 1A). RA or calcitriol significantly reduced cyclophosphamide treatment-induced primordial follicle loss, with the most effective doses of 25 mg/kg RA and 50 ng/kg calcitriol (Figure 1B,C). The co-treatment of RA and calcitriol (definition as Vit) further reduced cyclophosphamide treatment-induced primordial follicle loss (Figure 1D,E). A relatively weak protective effect of Vit was observed in the doxorubicin-treated group (Figure 1B–F).

We also examined DDX4 (DEAD-box helicase 4), a germ cell marker protein. Compared with the control, cyclophosphamide treatment resulted in a significant decrease in DDX4 protein levels, likely due to the reduction in primordial follicle numbers (Figure 1G and Figure S4). This was partially reversed by Vit treatment (Figure 1G). Consistent with the results of RA or calcitriol (Figures S2 and S5), Vit treatment significantly decreased the number of growing follicles compared with control (Figure 1D–F). Thus, the subsequent experiments focused on the mechanism of Vit in preventing cyclophosphamide treatment-induced mouse primordial follicle loss.

### 3.2. Transcriptomic Analysis Reveals Regulatory Pathways Involved in Vit Effects on the Ovaries from Cyclophosphamide-Treated Neonatal Mice

**Figure 2:**
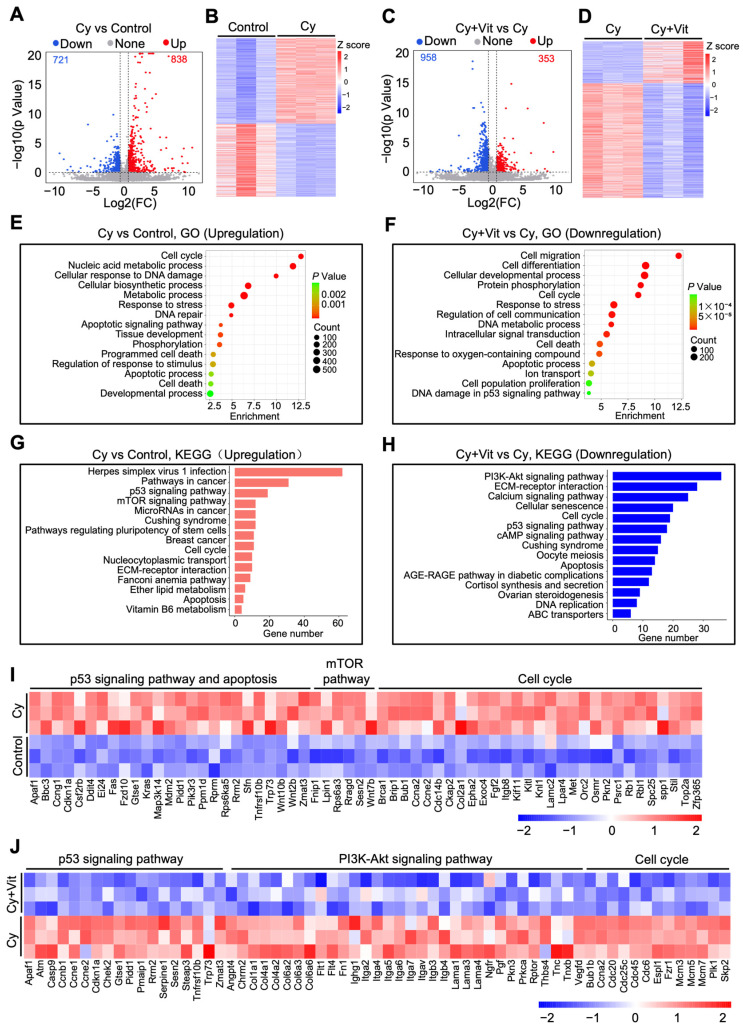
Effects of cyclophosphamide and Vit on the ovarian transcriptome in neonatal mice. Cyclophosphamide (Cy; 75 mg/kg) was intraperitoneally injected into the mice on 5 dpp. At the same time, Vit (25 mg/kg RA + 50 ng/kg calcitriol) was intraperitoneally injected into the mice on 3 and 5 dpp. Equal doses of PBS or DMSO were intraperitoneally injected into the mice as the corresponding controls. Ovaries were collected from mice on 6 dpp for RNA-seq: (**A**,**B**) Volcano plot (**A**) and heatmap (**B**) show DEGs in ovaries from the cyclophosphamide group compared with control, *n =* 3, and each from 12 ovaries. (**C**,**D**) Volcano plot (**C**) and heatmap (**D**) show DEGs in ovaries from the cyclophosphamide + Vit group compared with the cyclophosphamide group. (**E**,**F**) GO analysis of upregulated DEGs from the cyclophosphamide group compared with the control (**E**) and of downregulated DEGs from the cyclophosphamide + Vit group compared with the cyclophosphamide group. (**G**,**H**) KEGG analysis of upregulated DEGs from the cyclophosphamide group compared with the control (**G**) and of downregulated DEGs from cyclophosphamide + Vit group compared with the cyclophosphamide group (**H**). (**I**) The heatmap shows the differential expression of a set of upregulated DEGs involved in different processes between the control and cyclophosphamide groups. (**J**) The heatmap shows the differential expression of a set of downregulated DEGs involved in different processes between the cyclophosphamide + Vit and cyclophosphamide groups. Cy, cyclophosphamide; Vit, RA + calcitriol.

We further studied the effects of Vit on transcriptomic changes in the ovaries of cyclophosphamide-treated neonatal mice. The volcano plot and heatmap analyses revealed that 1559 differentially expressed genes (DEGs, 838 upregulation and 721 downregulation) were present in the ovaries of the cyclophosphamide treatment group compared with the control (Figure 2A,B). The upregulated DEGs were mainly related to the p53 pathway, apoptosis, DNA damage and repair, the mTOR pathway, oxidative phosphorylation, cell proliferation, and tissue development (Figure 2E,G,I), and the downregulated DEGs were mainly related to ribosome metabolism, ATP production, and the mitochondrial electron transport chain (Figure S6A,B,E). Compared with the cyclophosphamide treatment group, the co-treatment of cyclophosphamide and Vit resulted in 1311 DEGs (353 upregulation and 958 downregulation, Figure 2C,D). The upregulated DEGs were associated with the TGF-β signaling pathway, the TNF signaling pathway, and immune response processes (Figure S6C,D), while the downregulated DEGs were mainly associated with the PI3K/Akt signaling pathway, the p53 signaling pathway, apoptosis, and cell cycle regulation (Figure 2F,H,J).

**Figure 3:**
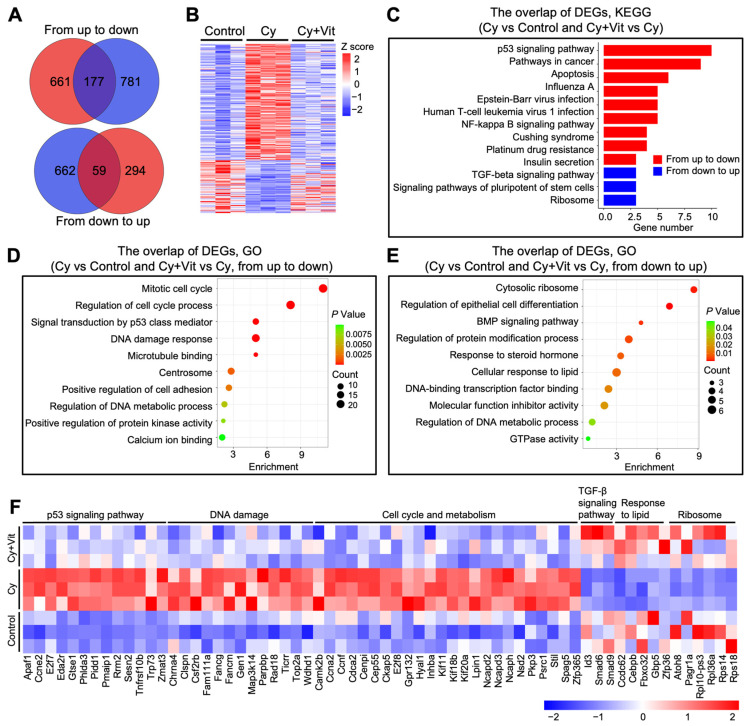
Effects of Vit on the transcriptome levels in the ovaries from cyclophosphamide-treated neonatal mice: (**A**) Venn diagram shows the overlap of DEGs in ovaries from the control, cyclophosphamide, and cyclophosphamide + Vit groups. From up to down: the overlap of upregulated DEGs in the cyclophosphamide group compared with the control, and downregulated DEGs in the cyclophosphamide + Vit group compared with the cyclophosphamide group. From down to up: the overlap of downregulated DEGs in the cyclophosphamide group compared with the control and upregulated DEGs in the cyclophosphamide + Vit group compared with the cyclophosphamide group. (**B**,**C**) Heatmap (**B**) and KEGG analysis (**C**) show the overlapping DEGs from the control, cyclophosphamide, and cyclophosphamide + Vit groups in the Venn diagram. (**D**,**E**) GO analysis of the overlapping DEGs in the Venn diagram, from up to down (**D**) and from down to up (**E**). (**F**) The heatmap shows a set of overlapping DEGs in the Venn diagram involved in different biological processes. Cy, cyclophosphamide; Vit, RA + calcitriol.

Further Venn diagram and heatmap analysis showed the overlap of these DEGs (Figure 3A,B). Cyclophosphamide treatment upregulated 838 DEGs, among which 177 DEGs were downregulated by Vit (from up to down). These 177 DEGs were mainly enriched in the p53 signaling pathway, DNA damage response, and apoptotic regulation pathways (Figure 3C,D,F). By contrast, cyclophosphamide treatment downregulated 721 DEGs, among which 59 DEGs were upregulated by Vit (from down to up). These 59 DEGs were mainly enriched in the TGF-β signaling pathway, response to lipid, and ribosome (Figure 3C,E,F). Based on the above observations, we hypothesize that Vit reduces cyclophosphamide treatment-induced primordial follicle loss by inhibiting DNA damage and apoptosis.

### 3.3. Vit Partially Reverses Cyclophosphamide Treatment-Induced Primordial Follicle Oocyte Transcriptional Activity

**Figure 4:**
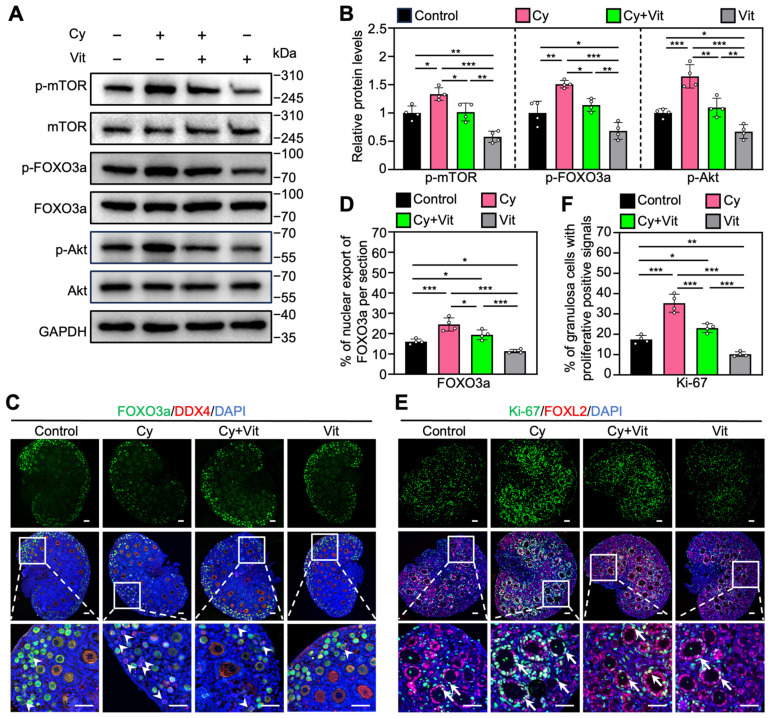
Effects of Vit on the PI3K/Akt pathway activation in the ovaries from cyclophosphamide-treated neonatal mice: (**A**,**B**) The comparison of p-mTOR, p-FOXO3a, and p-Akt protein levels by Western blot across various groups, *n* = 4, and each from 6–8 ovaries. (**C**,**D**) FOXO3a localization in primordial follicle oocyte cytoplasm (white arrowheads; **C**) and the comparison of FOXO3a nuclear export percentage in primordial follicle oocytes (**D**) across various groups, *n* = 4, and each from 5 sections of 1 ovary. (**E**,**F**) Ki-67 immunofluorescence stain (**E**) and the comparison of Ki-67-positive granulosa cell percentage (**F**) across various groups, *n* = 4, and each from 5 sections of 1 ovary. White arrows, growing follicles. Cy, cyclophosphamide; Vit, RA + calcitriol. The representative images were displayed. Scale bar, 50 µm. Bars indicate the mean ± SD. Data were analyzed by one-way ANOVA followed by Tukey’s multiple-comparison test. * *p* < 0.05, ** *p* < 0.01, and *** *p* < 0.001.

Consistent with previous studies [48,51,52], cyclophosphamide treatment increased the protein levels of p-mTOR, p-Akt, and p-FOXO3a (Figure 4A,B and Figure S7A), as well as the proportions of primordial follicle oocytes with FOXO3a nuclear export (Figure 4C,D) and of granulosa cells with Ki-67-positive signals (Figure 4E,F) compared with control. However, all of these cyclophosphamide treatment-induced increases were partially reversed by Vit (Figure 4). Vit treatment alone also reduced these phosphorylated protein levels, and the proportions of oocytes with FOXO3a nuclear export and granulosa cells with Ki-67 positive signaling compared with control (Figure 4). Therefore, Vit inhibited cyclophosphamide treatment-induced PI3K/Akt activation and FOXO3a nuclear export in primordial follicle oocytes, suggesting that Vit partially reverses cyclophosphamide treatment-induced primordial follicle oocyte transcriptional activity.

### 3.4. Vit Partially Reverses Cyclophosphamide Treatment-Induced Oxidative Stress and DNA Damage

**Figure 5:**
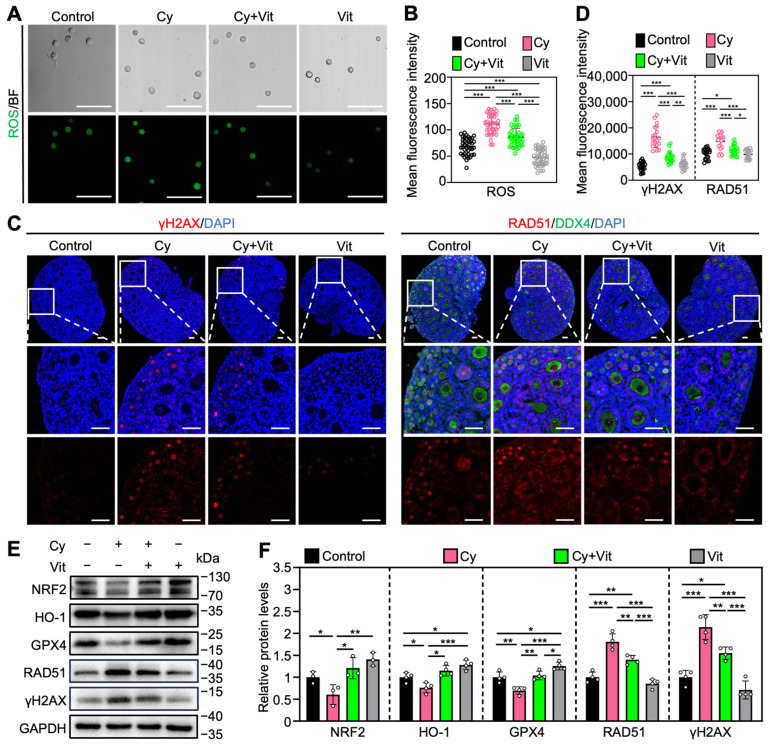
Effects of Vit on oxidative stress and DNA damage in ovaries from cyclophosphamide-treated neonatal mice: (**A**,**B**) The ROS staining (green; **A**) and the relative fluorescence intensity (**B**) across various groups, *n* = 40 primordial follicle oocytes from 8 ovaries. BF, bright field. (**C**,**D**) γ-H2AX and RAD51 immunofluorescence staining (red; **C**) and the relative fluorescence intensity (**D**) across various groups. *n* = 20, and each from 5 to 10 primordial follicle oocytes within one ovarian section. (**E**,**F**) The comparison of NRF2, HO-1, GPX4, RAD51, and γ-H2AX protein levels by Western blot across various groups, *n* = 4, and each from 6 to 8 ovaries. Cy, cyclophosphamide; Vit, RA + calcitriol. The representative images are displayed. Scale bar, 50 µm. Bars indicate the mean ± SD. Data were analyzed by one-way ANOVA followed by Tukey’s multiple-comparison test. * *p* < 0.05, ** *p* < 0.01, and *** *p* < 0.001.

Consistent with previous studies [20,53,54,55,56,57,58], cyclophosphamide treatment increased ROS levels in the primordial follicle oocytes (Figure 5A,B) and the protein levels of γ-H2AX and RAD51 in the ovaries (Figure 5C–F and Figure S7B), while decreasing the protein levels of NRF2, HO-1, and GPX4 in the ovaries compared with control (Figure 5E,F). However, all of these cyclophosphamide treatment-induced changes were partially reversed by Vit (Figure 5). Vit treatment alone also reduced ROS levels in primordial follicle oocytes (Figure 5A,B) and increased the protein levels of HO-1 and GPX4 in the ovaries compared with control (Figure 5E,F). These findings indicate that Vit treatment enhances the antioxidant capacity and restores redox homeostasis in the neonatal mouse ovary. Therefore, Vit partially reverses cyclophosphamide treatment-induced oxidative stress and DNA damage.

### 3.5. Vit Partially Reverses Cyclophosphamide Treatment-Induced Primordial Follicle Apoptosis

**Figure 6:**
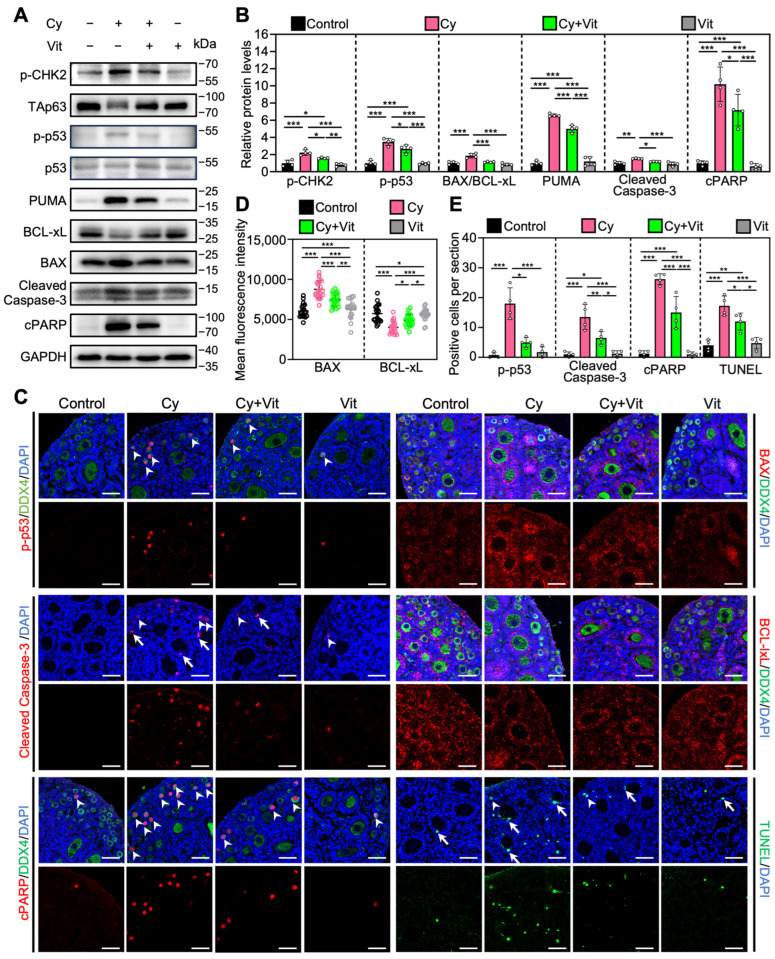
Effects of Vit on primordial follicle oocyte apoptosis in the ovaries from cyclophosphamide-treated neonatal mice: (**A**,**B**) The comparison of p-CHEK2, TAp63α, p53, PUMA, BCL-xL, BAX, Cleaved Caspase-3, and cPARP protein levels by Western blot across various groups, *n* = 4, and each from 6 to 8 ovaries. Cyclophosphamide treatment induced a mobility shift of TAp63 that was absent in the control. (**C**) Immunofluorescence staining of p-p53 (red), BAX (red), Cleaved Caspase-3 (red), BCL-xL (red), and TUNEL (green) across various groups. White arrowheads, primordial follicle oocytes. White arrows, granulosa cells. Scale bars, 50 µm. (**D**) Relative fluorescence intensity of BAX and BCL-xL. *n* = 20, and each from 5 to 10 primordial follicle oocytes within one ovarian section. (**E**) The comparison of p-p53, Cleaved Caspase-3, cPARP, and TUNEL-positive cell number per ovarian section across various groups, *n* = 4, and each from 5 sections of 1 ovary. Cy, cyclophosphamide; Vit, RA + calcitriol. The representative images were displayed. Bars indicate the mean ± SD. Data were analyzed by one-way ANOVA followed by Tukey’s multiple-comparison test. * *p* < 0.05, ** *p* < 0.01, and *** *p* < 0.001.

Consistent with previous studies [54,59,60], cyclophosphamide treatment increased the levels of pro-apoptotic proteins (p-CHK2, p-p53, PUMA, BAX, Cleaved Caspase-3, and cPARP) and the number of cells with TUNEL-positive signals, while decreasing the levels of the anti-apoptotic protein BCLxL in the ovaries compared with control (Figure 6 and Figure S8). In addition, cyclophosphamide treatment induced TAp63 activation, as indicated by a mobility shift that was absent in the control group (Figure 6A). Immunofluorescence analysis showed that cyclophosphamide treatment increased p-p53 levels (Figure 6C–E) and decreased TAp63 levels (Figure S9A,B) in the oocyte nuclei of primordial follicles compared with the control. However, all of these cyclophosphamide treatment-induced changes were partially reversed by Vit (Figure 6). Therefore, Vit partially reverses cyclophosphamide treatment-induced primordial follicle apoptosis.

### 3.6. Vit Preserves Fertility in Cyclophosphamide-Treated Mice

**Figure 7:**
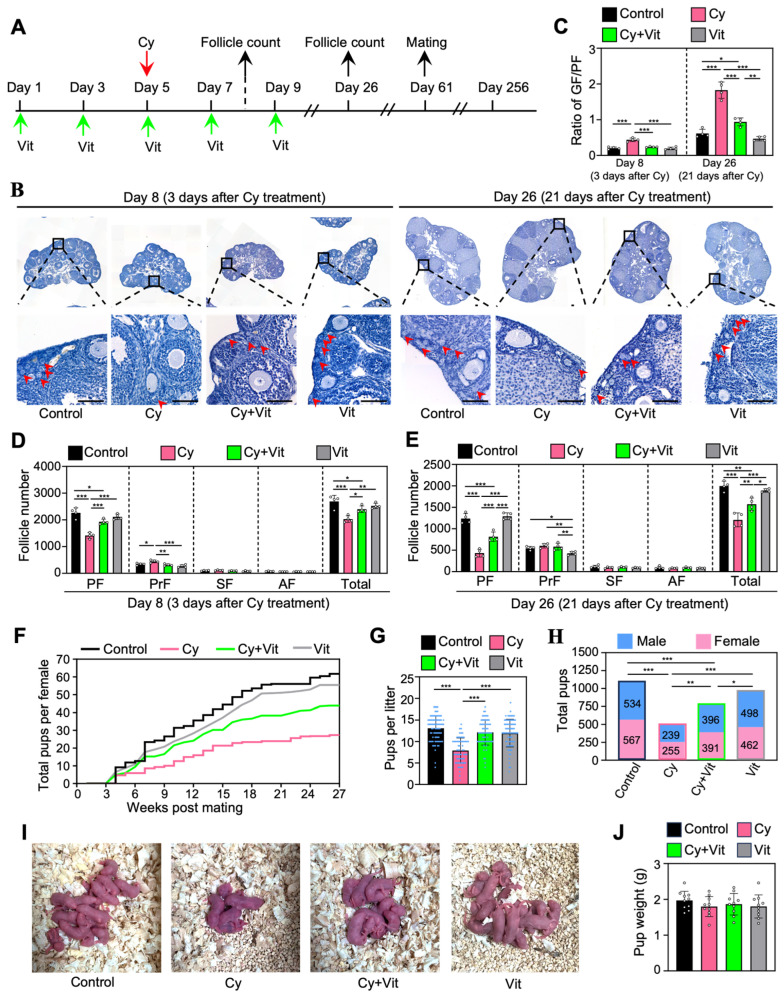
Effects of Vit on fertility in cyclophosphamide-treated adolescent mice: (**A**) Experimental schema. Adolescent mice (mice on 21 dpp as day 1) were intraperitoneally injected with cyclophosphamide (75 mg/kg) on day 5 (red arrow). At the same time, Vit (25 mg/kg RA + 50 ng/kg calcitriol) was intraperitoneally injected into the mice on days 1, 3, 5, 7, and 9 (green arrows). Equal doses of PBS or DMSO were intraperitoneally injected into the mice as the corresponding controls. Some mice were sacrificed on day 8 and 26 (black arrows) for ovarian follicle counting (**B**–**E**), and the remaining mice were mated with fertility-proven males for the fertility test on day 61 (black arrow) until day 256 (**F**–**J**). (**B**–**E**) Ovarian morphological comparison (**B**), the ratio of growing follicles (primary, secondary, and antral follicles) to dormant follicles (primordial follicles) across various groups (**C**), and the number of follicles at different stages across various groups on day 8 (**D**) and day 26 (**E**), *n* = 4, and each from 3 ovaries. The ovarian sections were hematoxylin-stained. Red arrowheads, primordial follicles. PF, primordial follicle; PrF, primary follicle; SF, secondary follicle; AF, antral follicle. Scale bar: 100 µm. (**F**–**H**) The comparison of the cumulative number of pups per mouse (**F**), the number of pups per litter (**G**), and the total number of pups (**H**) across various groups. (**I**) The comparison of mean body weight in pups per litter across various groups. (**J**) Representative images of pups in one litter. Cy, cyclophosphamide; Vit, RA + calcitriol. The representative images were displayed. Bars indicate the mean ± SD. Data were analyzed by one-way ANOVA followed by Tukey’s multiple-comparison test. * *p* < 0.05, ** *p* < 0.01, and *** *p* < 0.001.

To investigate the effect of Vit on the fertility in cyclophosphamide-treated mice, we intraperitoneally injected adolescent mice with cyclophosphamide (day 5), and/or with Vit (days 1, 3, 5, 7, and 9) before and after cyclophosphamide treatment (Figure 7A). Ovaries were collected for follicle counting on day 8 (3 days post-cyclophosphamide treatment) and day 26 (Figure 7A). The remaining female mice were mated with fertility-proven males for the fertility test on day 61 after the start of the experiment (Figure 7A).

Consistent with previous studies [52,61], a single injection of cyclophosphamide significantly decreased primordial follicle numbers on day 8 and further decreased the number on day 26 (Figure 7B–E), but had no obvious effect on the ovarian morphology compared with control (Figure S10). Consistent with this, cyclophosphamide treatment increased the ratio of growing/primordial follicles compared with control (Figure 7C). However, all of these cyclophosphamide treatment-induced changes were partially reversed by Vit (Figure 7B–E). Cyclophosphamide significantly increased the number of primary follicles on day 8 compared with control, but had no effect on the number of growing follicles on day 26 compared with control (Figure 7B–E), possibly because cyclophosphamide treatment-induced growing follicles failed to develop normally.

Consistent with previous studies [48,49,59], cyclophosphamide also significantly decreased the cumulative number of pups per mouse, the number of pups per litter, and the total number of pups compared with control (Figure 7F–H). However, Vit treatment partially reversed these cyclophosphamide-induced decreases in fertility. All groups of neonatal mice had no obvious malformations and no weight differences (Figure 7I,J). Therefore, Vit preserves fertility in cyclophosphamide-treated mice by preserving the reserve of primordial follicles.

### 3.7. Vit Preserves the Primordial Follicle Reserve in Cyclophosphamide-Treated MCF-7 Tumor-Bearing Mice

**Figure 8:**
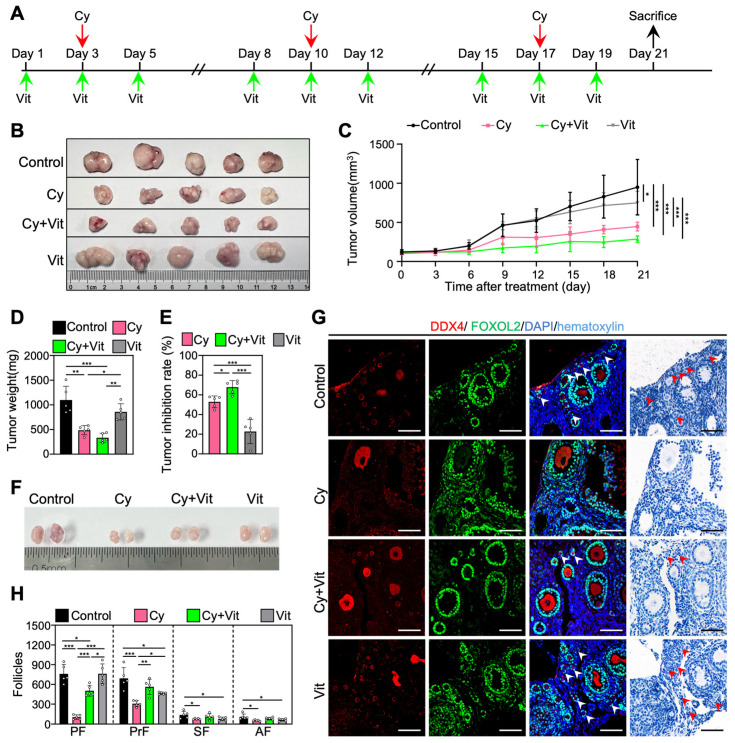
The antitumor effects of Vit on cyclophosphamide in MCF-7 tumor-bearing mice: (**A**) Experimental schema. MCF-7 tumor-bearing mice were intraperitoneally injected with cyclophosphamide (75 mg/kg) on days 3, 10, and 17 (red arrows), and with Vit (25 mg/kg RA + 50 ng/kg calcitriol) on days 1, 3, 5, 8, 10, 12, 15, 17, and 19 (green arrows). Equal doses of PBS or DMSO were intraperitoneally injected into the mice as the corresponding controls. Mice were sacrificed on day 21 (black arrow), and the ovaries and tumors were collected for tumor inhibition analysis (**B**–**E**) and follicle counting (**F**,**G**). (**B**–**E**) Representative tumor images (**B**), tumor growth curves (**C**), tumor weights (**D**), and tumor inhibition rates (**E**) across various groups, *n =* 5, and each from one mouse. (**F**–**H**) Ovarian size comparison (**F**), ovarian morphological comparison (**G**), and the number of follicles (**H**) at different stages across various groups, *n* = 5, and each from 2 ovaries. Red arrowheads, primordial follicles. PF, primordial follicle; PrF, primary follicle; SF, secondary follicle; AF, antral follicle. Scale bar: 100 µm. Cy, cyclophosphamide; Vit, RA + calcitriol. The representative images were displayed. Bars indicate the mean ± SD. Data were analyzed by one-way ANOVA followed by Tukey’s multiple-comparison test. * *p* < 0.05, ** *p* < 0.01, and *** *p* < 0.001.

We established an MCF-7 tumor-bearing mouse model to investigate whether Vit can protect the primordial follicle pool during cyclophosphamide treatment. The 8-week-old BALB/c-Nude mice were subcutaneously injected with MCF-7 cells into the left mammary fat pad. When the tumor volume was close to 100 mm^3^ (day 1), MCF-7 tumor-bearing mice were injected intraperitoneally with cyclophosphamide three times (days 3, 10, and 17), and/or with Vit before and after each cyclophosphamide treatment (Figure 8A). All mice were euthanized on day 21 after the start of the experiment for ovary and tumor collection (Figure 8A).

Consistent with previous studies [62,63], three doses of cyclophosphamide injection significantly decreased the volume and weight of tumors (Figure 8B–E and Figure S11), the size of ovaries (Figure 8F), and the number of primordial, primary, secondary, and antral follicles (Figure 8G,H) compared with control. However, Vit partially reversed the cyclophosphamide-induced decrease in the number of primordial and growing follicles (Figure 8G,H). Vit treatment alone also demonstrated modest antitumor activity and further increased the tumor inhibition rate of cyclophosphamide treatment in MCF-7 tumor-bearing mice (Figure 8B–E), consistent with previous studies on MCF-7 cells [64,65]. Therefore, Vit preserves the primordial follicle reserve in cyclophosphamide-treated MCF-7 tumor-bearing mice.

## 4. Discussion

**Figure 9:**
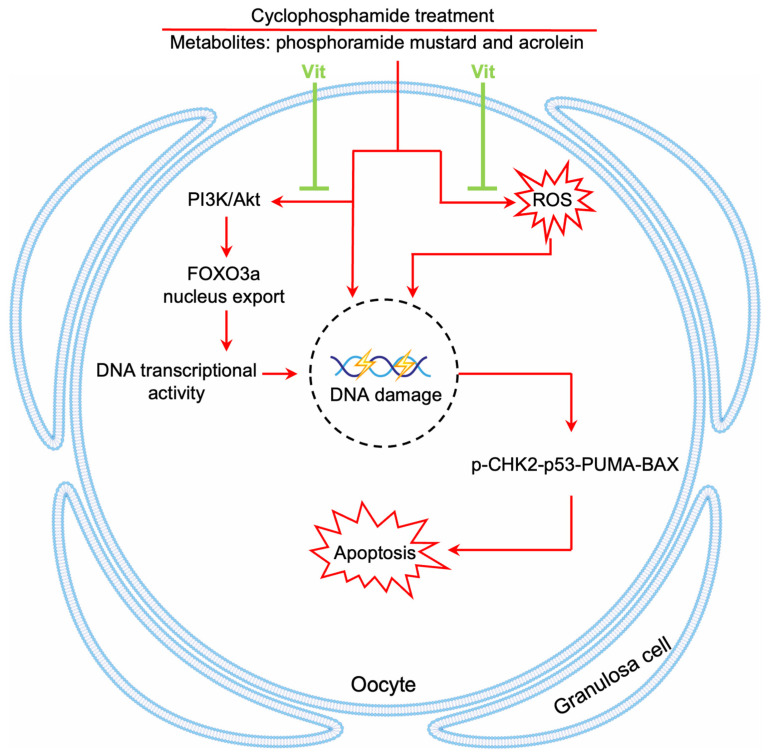
A model depicting Vit-mediated protection of primordial follicles from cyclophosphamide treatment-induced apoptosis. RA and calcitriol co-treatment (Vit) protects mouse primordial follicles from cyclophosphamide treatment-induced apoptosis by inhibiting cyclophosphamide treatment-induced oocyte transcriptional activity and enhancing antioxidant capacity.

Chemotherapy causes a dramatic decline in the ovarian reserve of cancer patients, ultimately leading to POI and even infertility [1]. In the present study, RA and calcitriol reduced cyclophosphamide and doxorubicin treatment-induced primordial follicle depletion in neonatal mouse ovaries. Furthermore, RA and calcitriol co-treatment preserved fertility in cyclophosphamide-treated mice without impairing cyclophosphamide’s antitumor efficacy in MCF-7 tumor-bearing mice. Therefore, the mechanism of RA and calcitriol involves enhancing antioxidant capacity and inhibiting cyclophosphamide treatment-induced DNA transcriptional activity, leading to reduced binding of cyclophosphamide’s active metabolites to primordial follicle oocyte DNA (Figure 9).

Cyclophosphamide treatment produces the active metabolite phosphoramide mustard in the liver [66]. Phosphoramide mustard forms cross-links with DNA and interferes with DNA replication and transcription, ultimately leading to cell cycle arrest and apoptosis in rapidly dividing cancer cells [9]. Postnatal mammalian oocytes are arrested at the diplotene stage of meiosis prophase I but nevertheless execute widespread transcriptional changes, and the oocyte nucleus is very large with a loose chromatin structure [67,68,69]. Thus, oocytes are sensitive to chemotherapy drugs [70]. Although the oocytes within primordial follicles are in a relatively quiescent state, they are more sensitive to chemotherapy drugs due to the lack of both DNA repair ability and antioxidant capacity in the oocytes within growing follicles [71,72]. In the present study, the injection of cyclophosphamide or doxorubicin into neonatal mice led to the depletion of primordial follicles by upregulating the DNA damage response and the p53-dependent apoptotic pathway in oocytes. These results are consistent with previous studies [20,59].

Cyclophosphamide treatment decreased ovarian reserve mainly by inducing primordial follicle apoptosis in neonatal mice [73,74]. Cyclophosphamide treatment increases the phosphorylation levels of PI3K/Akt and FOXO3a in the ovaries of neonatal mice, as shown in our study and previous studies [48,51]. FOXO3a, a nuclear transcriptional repressor, maintains primordial follicle oocytes’ dormancy by inhibiting their transcriptional activity [75]. Cyclophosphamide treatment induced FOXO3a phosphorylation and then promoted FOXO3a translocation from the nucleus to the cytoplasm, resulting in an increase in the transcriptional activity in primordial follicle oocytes. The increased transcriptional activity in primordial follicle oocytes could further promote the binding of cyclophosphamide’s active metabolites to DNA, thereby promoting primordial follicle apoptosis. RA and calcitriol could inhibit PI3K/Akt activity and reduce FOXO3a phosphorylation levels and nuclear export in primordial follicle oocytes, resulting in decreased transcriptional activity in primordial follicle oocytes. Further studies from our laboratory indicated that both RA and calcitriol could interact with p85α by binding to their respective receptors to inhibit the PI3K/Akt signaling pathway (unpublished data). Thus, RA and calcitriol inhibit cyclophosphamide treatment-induced DNA transcriptional activity, possibly leading to a reduction in oxidative stress and primordial follicle apoptosis by decreasing the binding of cyclophosphamide’s active metabolites to primordial follicle oocyte DNA [9,76].

The metabolic products of cyclophosphamide, particularly acrolein, generate excessive ROS and impair the antioxidant defense system (GSH depletion), disrupting the redox homeostasis [77]. In the present study, cyclophosphamide treatment increased oxidative stress by increasing ROS levels in the oocytes of primordial follicles, consistent with previous studies in ovaries [78,79]. Cyclophosphamide treatment also increased oxidative stress by decreasing the protein levels of NRF2, HO-1, and GPX4 in neonatal mouse ovaries, consistent with previous studies in various tissues [80,81,82,83]. RA combined with chemotherapeutic agents is usually applied for the prevention and treatment of different types of cancers, particularly acute promyelocytic leukemia (APL) [84]. One of the mechanisms is that RA reduces NRF2 activity through RARα to increase the susceptibility to electrophiles and oxidative stressors in cancer cells [85]. However, RA and calcitriol could reduce ROS production and enhance antioxidant enzyme activities in bovine oocytes [34,86] and goat granulosa cells [42,87]. RA could reduce doxorubicin-induced cardiomyocyte apoptosis by inhibiting ROS generation and activating the antioxidant defense system [44,88]. Similarly, calcitriol has been shown to reduce hyperosmotic stress-induced cytotoxicity in human corneal epithelial cells by reducing intracellular ROS generation and activating NRF2-antioxidant signaling [89]. Consistent with the above studies, RA and calcitriol reversed the increase in ROS levels and decrease in antioxidant capacity induced by cyclophosphamide treatment, resulting in a reduction in primordial follicle apoptosis in neonatal mouse ovaries.

RA and calcitriol are used to treat leukemia [84] and breast cancer [90], respectively. We found that RA and calcitriol protected mouse primordial follicles from cyclophosphamide treatment-induced apoptosis. The toxic dose of RA in mice is 40–80 mg/kg [91], while the intraperitoneal injection of 4 μg/kg calcitriol in mice has no toxicity [92]. In the present study, 25 mg/kg RA and 50 ng/kg calcitriol were used to protect mouse primordial follicles from cyclophosphamide treatment-induced apoptosis. RA and calcitriol had no negative impact on mouse reproduction after observation for half a year (Figure 7). However, the efficacy and safety of RA and calcitriol require further clinical validation. Additionally, whether RA and calcitriol also protect against ovarian damage from other chemotherapeutics (e.g., cisplatin) requires further investigation.

In conclusion, our study indicates that RA and calcitriol partially reverse cyclophosphamide treatment-induced primordial follicle apoptosis by inhibiting cyclophosphamide treatment-induced transcriptional activity and enhancing antioxidant capacity (Figure 9). These findings suggest a potential non-invasive therapeutic approach for ovarian reserve protection during chemotherapy in female cancer patients.

## Data Availability

RNA-seq data have been submitted to the NCBI Sequence Read Archive (SRA) under accession number PRJNA1328229. All data supporting the findings of this study are available within the article and/or the Supplementary Information. Additional data related to this paper may be requested from the authors.

## Supplementary Materials

The following supporting information can be downloaded at: https://www.mdpi.com/article/10.3390/antiox15010068/s1, Figure S1: Single-cell RNA sequencing (scRNA-seq) analysis reveals VDR and RARs expression in neonatal mouse ovaries; Figure S2: Effects of retinoic acid or calcitriol on mouse primordial follicle activation; Figure S3: Effects of cyclophosphamide or doxorubicin treatment on mouse primordial follicle loss; Figure S4: Uncropped scans of Western blotting corresponding to Figure 1; Figure S5: Effects of Vit on the ovarian transcriptome in neonatal mice; Figure S6: Effects of cyclophosphamide and Vit on the ovarian transcriptome in neonatal mice; Figure S7: Uncropped scans of Western blots corresponding to Figure 4 and Figure 5; Figure S8: Uncropped scans of Western blots corresponding to Figure 6; Figure S9: Effects of Vit on primordial follicle apoptosis in ovaries from cyclophosphamide-treated neonatal mice; Figure S10: Effects of Vit on fertility in cyclophosphamide-treated adolescent mice; Figure S11: Effects of Vit on the antitumor of cyclophosphamide in MCF-7 tumor-bearing mice; Table S1: List of primary antibodies used in immune detection in this study.

## Abbreviations

The following abbreviations are used in this manuscript:

AF	Antral follicle
Akt	Protein kinase B
Bax	B-cell lymphoma 2-associated X
BCA	Bicinchoninic acid
BCLxL	B-cell lymphoma-extra large
Cal	Calcitriol
DAPI	4′,6-diamidino-2phenylindole
DCFH-DA	2′,7′-dichlorodihydrofluorescein diacetate
DDX4	DEAD-box helicase 4
DMEM	Dulbecco’s modified Eagle’s medium
DMSO	Dimethyl sulfoxide
dpp	Days postpartum
FBS	Fetal bovine serum
FOXO3a	Forkhead box O3a
GC	Granulosa cell
GF	Growing follicle
GPX4	Glutathione peroxidase
GSH	glutathione
HO-1	heme oxygenase-1
mTOR	Mammalian target of rapamycin
NRF2	Nuclear factor erythroid 2-related factor 2
PBS	Phosphate-buffered saline
PF	Primordial follicle
PI3K	Phosphoinositide 3-kinase
PrF	Primary follicle
PVDF	Polyvinylidene fluoride
RA	Retinoic acid
ROS	Reactive oxygen species
SF	Secondary follicle
Vit	Retinoic acid + calcitriol
