# Supplementary material for: Retinoic Acid and Calcitriol Protect Mouse Primordial Follicles from Cyclophosphamide Treatment-Induced Apoptosis
Source: Antioxidants (Basel). 2026 Jan 4;15(1):68. doi: 10.3390/antiox15010068 (PMC12837172; doi:10.3390/antiox15010068)
Supplement: Supplementary file 1 [file antioxidants-15-00068-s001.zip › antioxidants-4006569-Supplementary Materials.pdf]

Supplementary Materials

Retinoic acid and calcitriol protect mouse primordial follicles from cyclophosphamide treatment-induced apoptosis

Sihui He, Xiaodan Zhang, Wenjun Zhou, Ye Chen, Fengxin Liu, Weiyong Wang, Hongwei Wei, Yan Du, Meijia Zhang*

The Innovation Centre of Ministry of Education for Development and Diseases, the Second Affiliated Hospital, School of Medicine, South China University of Technology, Guangzhou 510006, China

Corresponding author: Meijia Zhang, E-mail: zhangmeijia@scut.edu.cn.

This file includes:

Supplementary Figures S1 to S11

Supplementary Table S1

Supplementary Figures

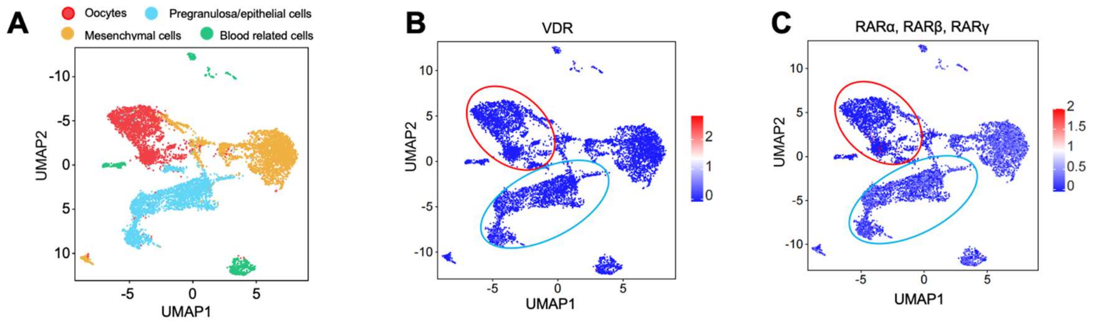

Figure S1. Single-cell RNA sequencing (scRNA-seq) analysis reveals VDR and RARs expression in neonatal mouse ovaries. A, uMAP plots of ovarian cells based on four cell types (oocytes, pregranulosa cells, mesenchymal cells and blood-related cells). B. uMAP visualization showing the expression distribution of VDR in pregranulosa cell and oocyte populations. C. uMAP visualization showing the expression distribution of RARs in pregranulosa cell and oocyte populations. The areas circled in red indicate the oocyte populations, while those circled in blue indicate the pregranulosa cell populations. The values were from published data (GSE263836).

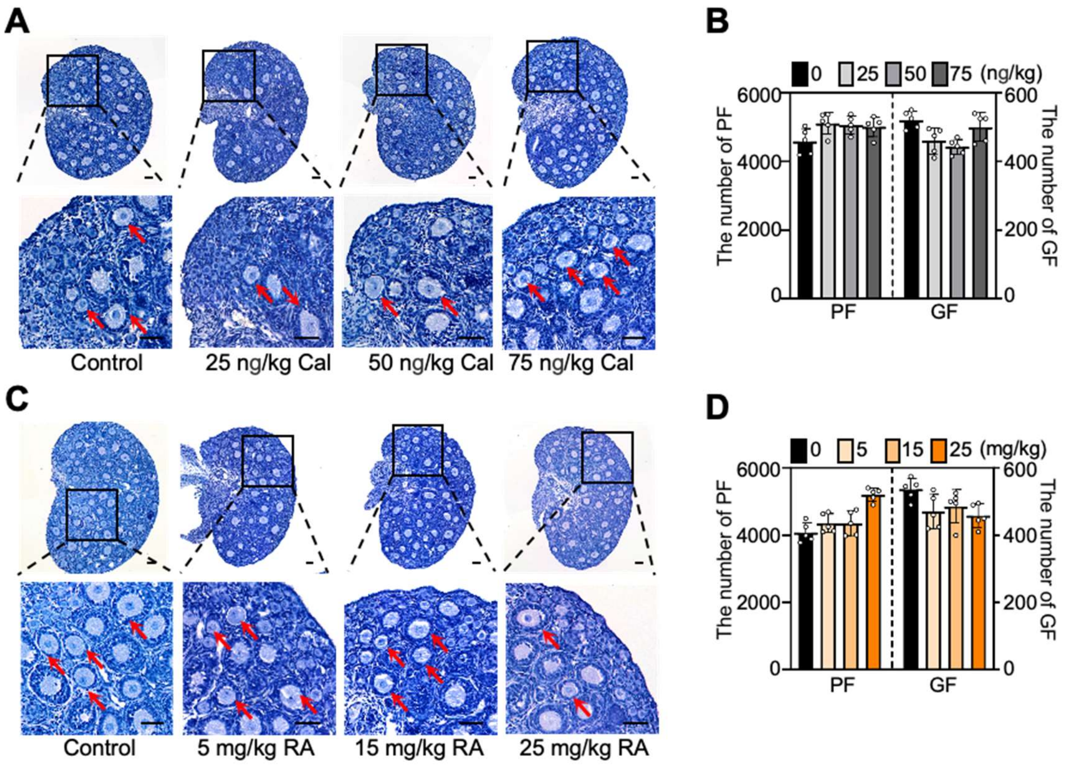

Figure S2. Effects of retinoic acid or calcitriol on mouse primordial follicle activation. Mice were intraperitoneally injected with retinoic acid (RA; 5, 15, 25 mg/kg) or calcitriol (Cal; 25, 50, 75 ng/kg) on 3, 5, and 7 dpp. Equal doses of DMSO were intraperitoneally injected into the mice as the corresponding controls. Ovaries were collected from mice on 8 dpp for follicle counting. **A-D,** Ovarian morphological comparison (**A**, **C**) and the number of primordial follicles (PF) and growing follicles (GF. **B**, **D**) across various groups, n=5, and each from 3 ovaries. The ovarian sections were hematoxylin-stained. Red arrows, growing follicles. Scale bars, 50 μm. The representative images were displayed. Bars indicate the mean ± SD. Data were analyzed by two-tailed unpaired t-test. *$p < 0.05$, **$p < 0.01$, and ***$p < 0.001$.

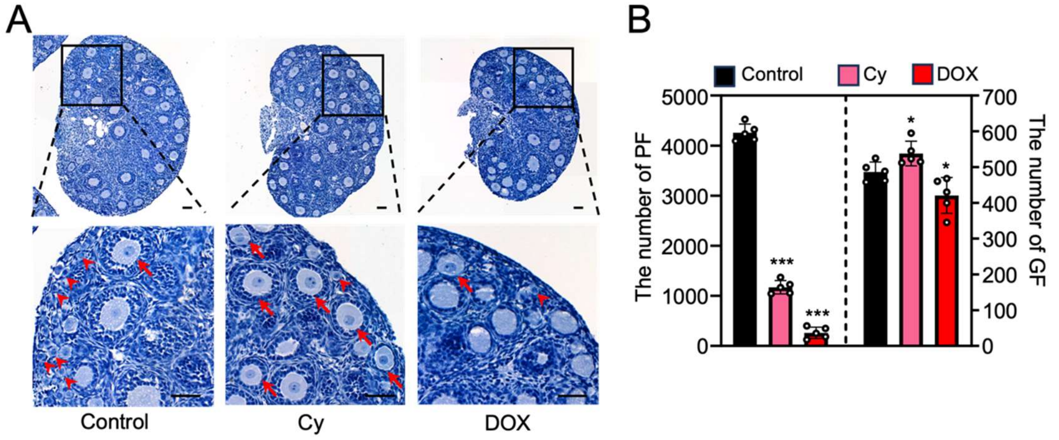

Figure S3. Effects of cyclophosphamide or doxorubicin treatment on mouse primordial follicle loss. 5 dpp mice were intraperitoneally injected with PBS, cyclophosphamide (Cy; 75 mg/kg), or doxorubicin (DOX; 10 mg/kg). Ovaries were collected from mice on 8 dpp for follicle counting. **A-B**, Ovarian morphological comparison (**A**) and the number of primordial follicles (PF) and growing follicles (GF. **B**) across various groups, n=5, and each from 3 ovaries. The ovarian sections were hematoxylin-stained. Red arrowheads, primordial follicles; red arrows, growing follicles. Scale bars, 50 µm. The representative images were displayed. Bars indicate the mean ± SD. Data were analyzed by two-tailed unpaired t-test. *$p < 0.05$, **$p < 0.01$, and ***$p < 0.001$.

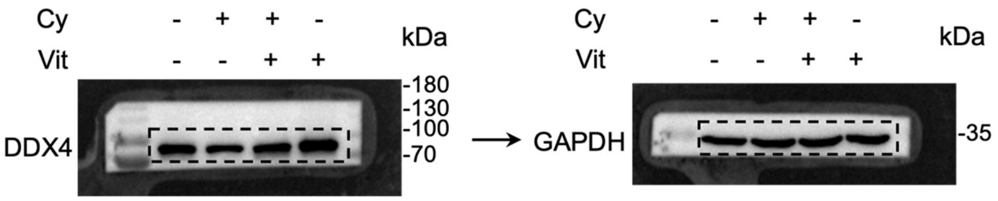

Figure S4. Uncropped scans of Western blotting corresponding to Fig. 1. The blots in the black dashed line boxes were used in Fig. 1G.

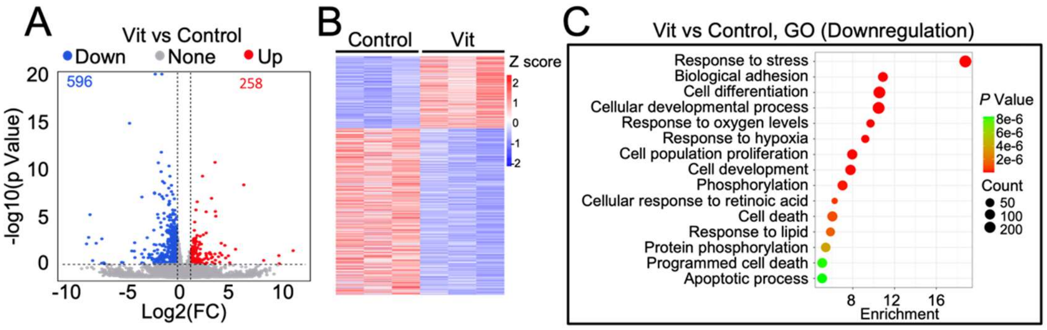

Figure S5. Effects of Vit on the ovarian transcriptome in neonatal mice. A-B, Volcano plot (**A**) and heatmap (**B**) show DEGs in ovaries from the Vit group compared with control, n = 3, and each from 12 ovaries. **C,** GO analysis of downregulated DEGs in ovaries from Vit and control groups. Cy, cyclophosphamide; Vit, RA + calcitriol.

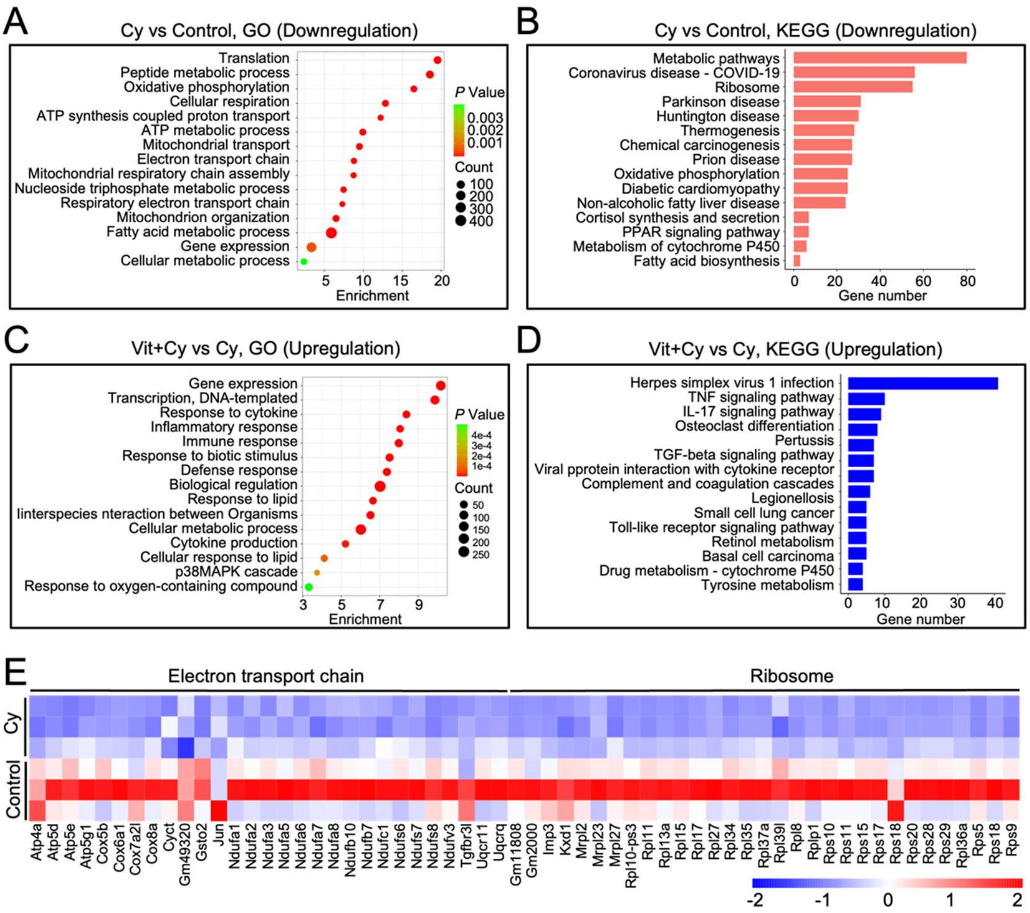

Figure S6. Effects of cyclophosphamide and Vit on the ovarian transcriptome in neonatal mice. A-B, GO analysis (**A**) and KEGG analysis (**B**) of downregulated DEGs in ovaries from the cyclophosphamide group compared with control, n = 3, and each from 12 ovaries. **C-D,** GO analysis (**C**) and KEGG analysis (**D**) of downregulated DEGs in ovaries from the cyclophosphamide + Vit group compared with cyclophosphamide group. **E,** Heatmap shows the differential expression of a set of downregulated DEGs involved in different processes between control and Cy groups. Cy, cyclophosphamide; Vit, RA + calcitriol.

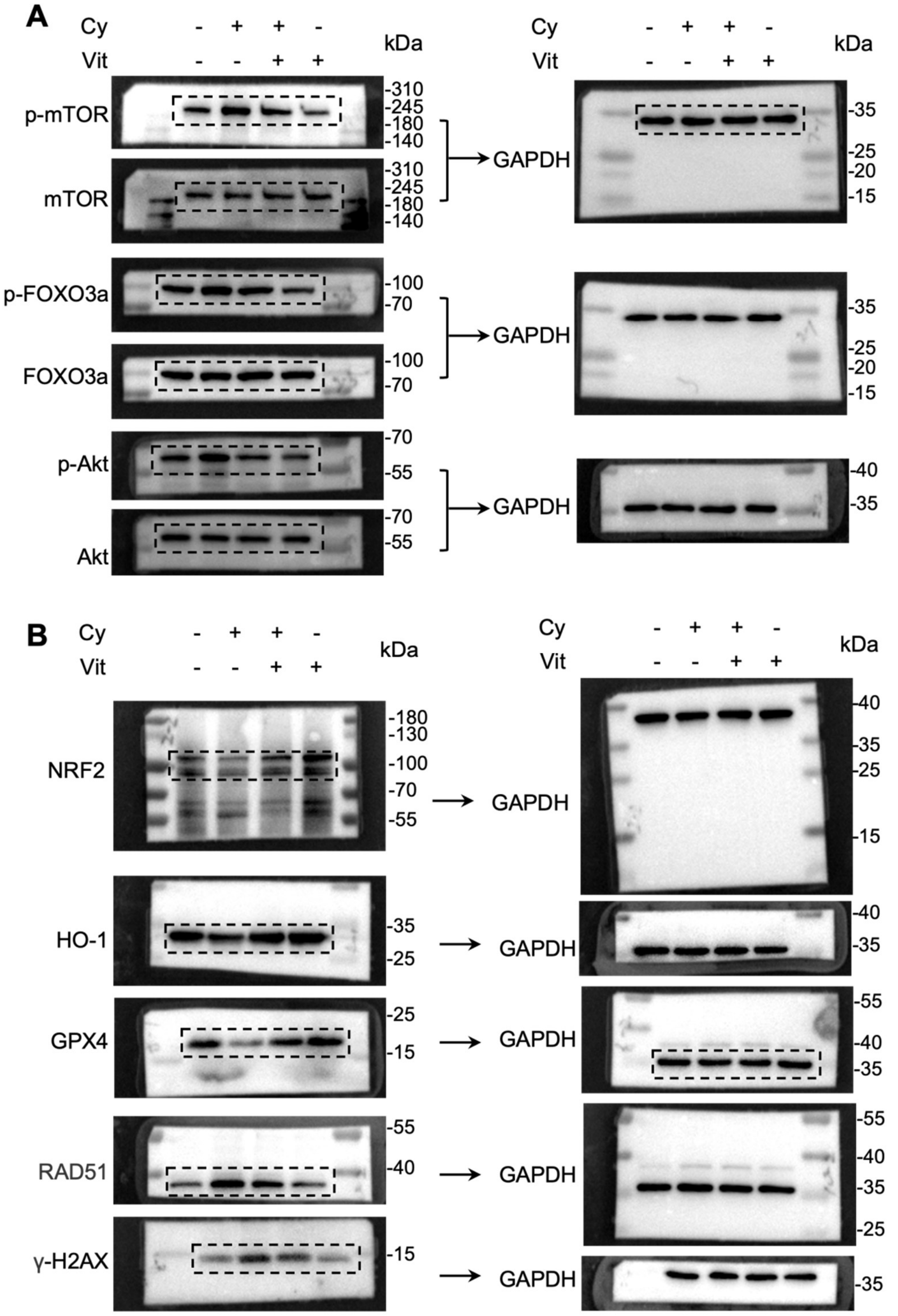

Figure S7. Uncropped scans of Western blots corresponding to Figures 4 and 5. A-B,

The blots in the black dashed line boxes were used in Figure 4A (**A**) and Figure 5E (**B**).

The black arrow points to the corresponding loading control. Cy, cyclophosphamide; Vit, RA + calcitriol.

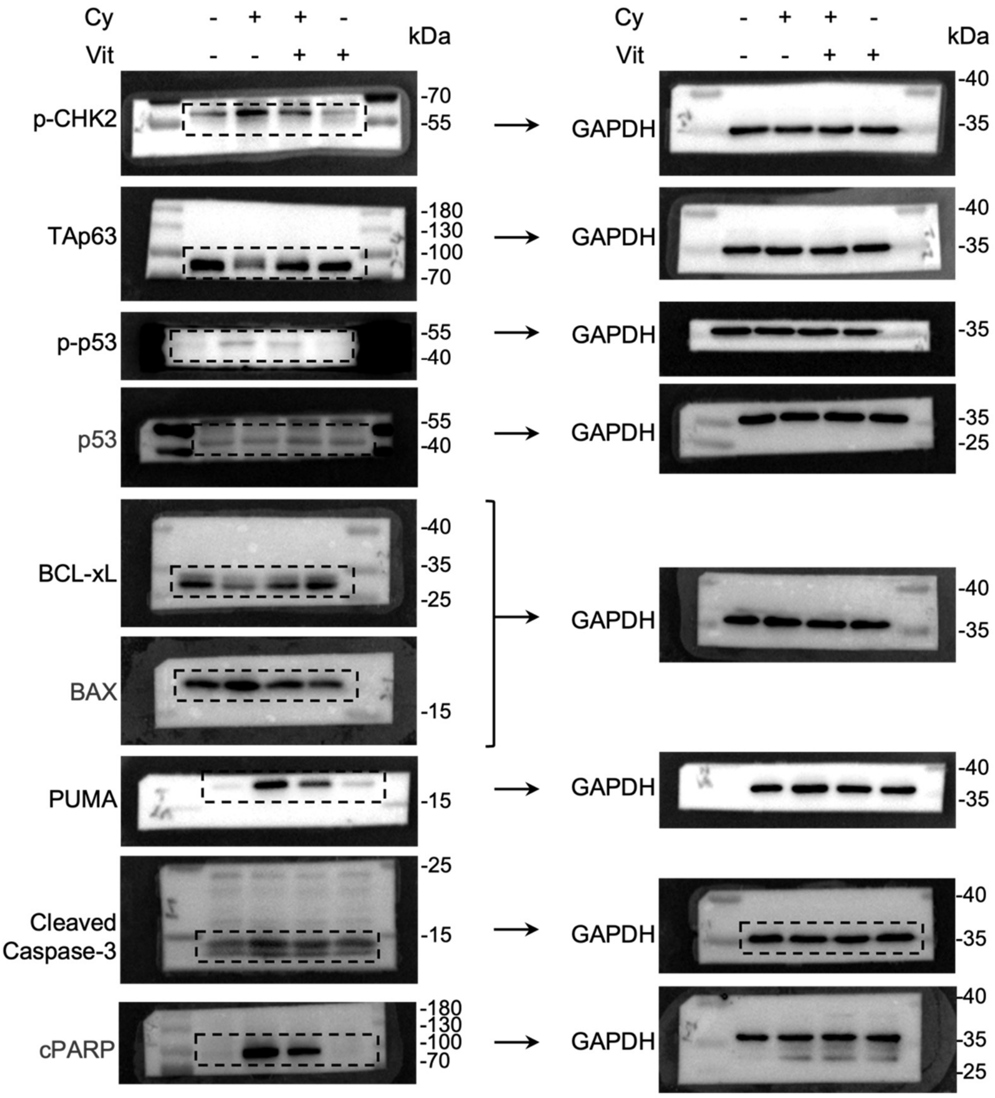

Figure S8. Uncropped scans of Western blots corresponding to Figures 6. The blots in the black dashed line boxes were used in Figure 6A. The black arrow points to the corresponding loading control.

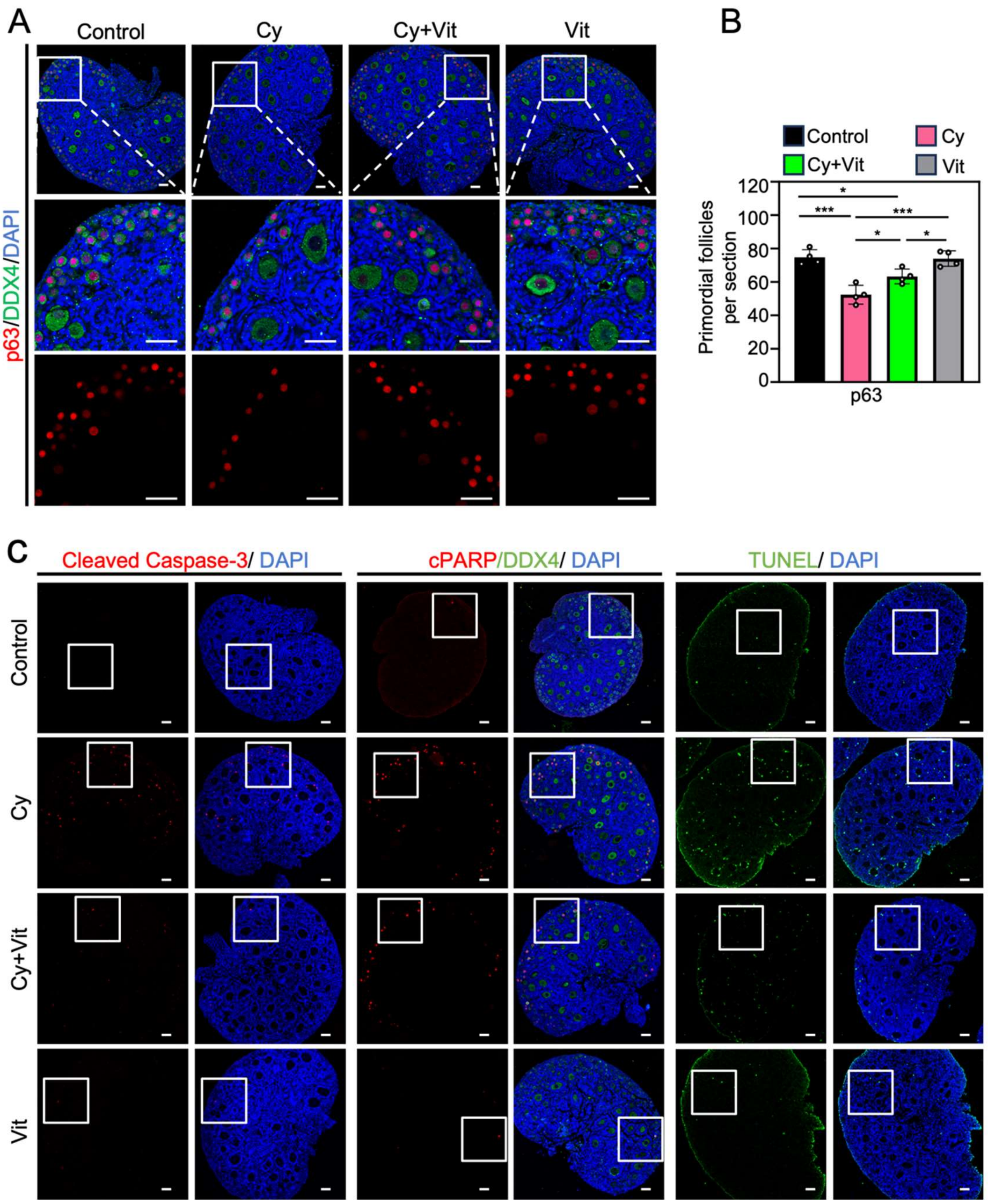

Figure S9. Effects of Vit on primordial follicle apoptosis in ovaries from cyclophosphamide-treated neonatal mice. A-B, Immunofluorescence staining (**A**) and statistical analysis (**B**) of p63 across various groups, n = 4, and each from 5 sections of 1 ovary. **C,** Immunofluorescence staining of Cleaved Caspase-3 (red), cPARP (red), and

TUNEL (green) in mouse ovaries across various groups. Scale bar: 50 μm. The amplified views of the boxed area are shown in Figure 6C. Cy, cyclophosphamide; Vit, RA + calcitriol. The representative images were displayed. Bars indicate the mean ± SD. Data were analyzed by one-way ANOVA followed by Tukey's multiple comparison test. *$p < 0.05$, **$p < 0.01$, and ***$p < 0.001$.

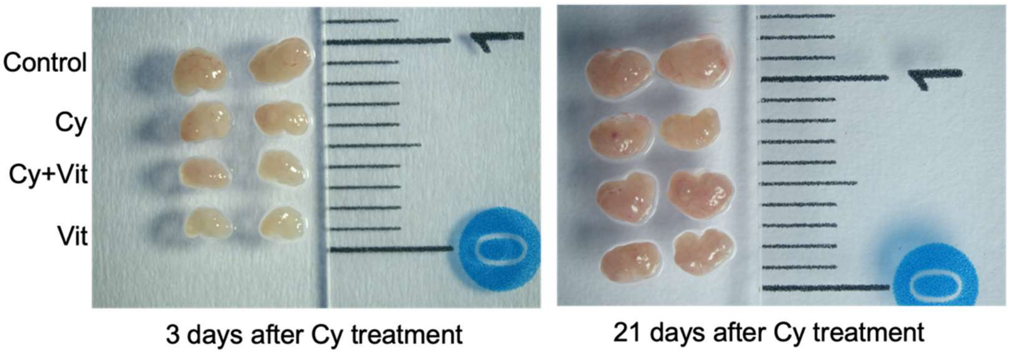

Figure S10. Effects of Vit on fertility in cyclophosphamide-treated adolescent mice. Ovarian size comparison across various groups. Cy, cyclophosphamide; Vit, RA + calcitriol.

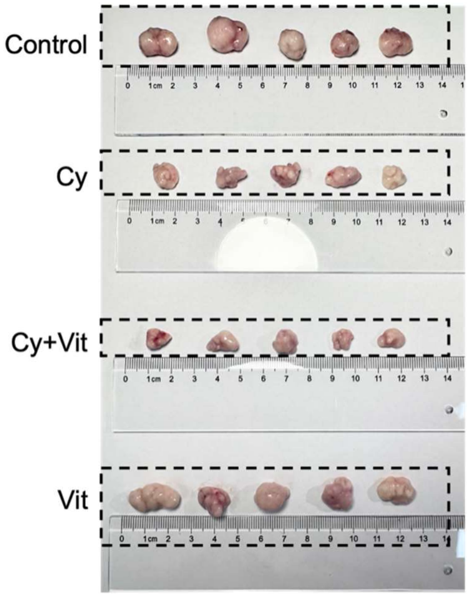

Figure S11. Effects of Vit on the anti-tumor of cyclophosphamide in MCF-7 tumor-bearing mice. Uncropped tumor images. The blots in the black dashed line boxes were used in Figure 8B. Cy, cyclophosphamide; Vit, RA + calcitriol.

Supplementary Table

Table S1. List of primary antibodies used in immune detection in this study.

Antibody	Catalog Code	Source	Host	Dilution	
				IF	WB
Ki-67	9129s	CST	Rabbit	1:200	—
Cleaved Caspase-3	9664	CST	Rabbit	1:50	1:1000
p-mTOR	2971	CST	Rabbit	-	1:1000
Phospho-p53-ser15	9284T	CST	Rabbit	1:200	—
p-FOXO3a	ab26649	Abcam	Rabbit	—	1:1000
Akt	4691	CST	Rabbit	—	1:1000
DDX4	ab27591	Abcam	Mouse	1:200	1:1000
mTOR	2972	CST	Rabbit	—	1:1000
p-Akt	4060	CST	Rabbit	—	1:1000
FOXO3a	12829	CST	Rabbit	1:100	1:1000
Phospho CHK2(Thr68)	2197T	CST	Rabbit	1:200	1:500
BAX	50599-2-Ig	Proteintech	Rabbit	—	1:1000
BCL-xL	ET1603-28	HUABIO	Rabbit	—	1:2000
p53	sc-126	Santa Cruz	Rabbit	1:100	—
PUMA	98672	CST	Rabbit	—	1:500
cPARP	9544T	CST	Rabbit	1:100	1:1000
γH2AX Alexa Fluor® 555	ab206900	Abcam	Rabbit	1:400	—
γH2AX	ET1602-2	HUABIO	Rabbit	—	1:2000
RAD51	ab133534	Abcam	Rabbit	1:200	1:1000
p63-alpha(D2K8X)	13109T	CST	Rabbit	1:200	1:1000
HO-1	ER1802-73	HUABIO	Rabbit	—	1:2000
GPX4	ET1706	HUABIO	Rabbit	—	1:2000
NRF2	12721T	CST	Rabbit	—	1:1000
GAPDH	2118	CST	Rabbit	—	1:1000
FOXL2	NB100-1277	Novus Biologicals	Goat	1:300	1:1000

IF: Immunofluorescence; WB: Western blotting.
